# An Object Model and Interaction Method for a Simulated Experience of Pottery on a Potter’s Wheel

**DOI:** 10.3390/s20113091

**Published:** 2020-05-29

**Authors:** Takafumi Matsumaru, Ami Morikawa

**Affiliations:** Graduate School of Information, Production and Systems (IPS), Waseda University, Kitakyushu 808-0135, Japan; mrkw@ruri.waseda.jp

**Keywords:** simulated experience system, pottery of potter’s wheel, object model, bowl shape, layered cylinder, interaction method, preliminary evaluation, maneuverability, visibility, satisfaction

## Abstract

This paper introduces an object model and an interaction method for a simulated experience of pottery on a potter’s wheel. Firstly, we propose a layered cylinder model for a 3D object of the pottery on a potter’s wheel. Secondly, we set three kinds of deformation functions to form the object model from an initial state to a bowl shape: shaping the external surface, forming the inner shape (deepening the opening and widening the opening), and reducing the total height. Next, as for the interaction method between a user and the model, we prepare a simple but similar method for hand-finger operations on pottery on a potter’s wheel, in which the index finger movement takes care of the external surface and the total height, and the thumb movement makes the inner shape. Those are implemented in the three-dimensional aerial image interface (3DAII) developed in our laboratory to build a simulated experience system. We confirm the operation of the proposed object model (layered cylinder model) and the functions of the prepared interaction method (a simple but similar method to actual hand-finger operations) through a preliminary evaluation of participants. The participants were asked to make three kinds of bowl shapes (cylindrical, dome-shaped, and flat-type) and then they answered the survey (maneuverability, visibility, and satisfaction). All participants could make something like three kinds of bowl shapes in less than 30 min from their first touch.

## 1. Introduction 

This paper introduces an object model and an interaction method for a simulated experience of pottery on a potter’s wheel. We set a design concept in which a user can directly superimpose his/her hand-finger on a three-dimensional (3D) image projected in the air without special conditions such as mounting equipment and environmental preparation. Through building the simulated experience system of pottery on a potter’s wheel as a case study, it would be examined whether the design concept contributes a lot to the human-system interaction in a certain practical application, along with the proposed object model (layered cylinder model) and the prepared interaction method (a simple but similar method to actual hand-finger operations). The system and technologies described in this paper differ from conventional studies and would contribute to in the following points: (1) the simulated experience system of pottery on a potter’s wheel constructed here enables a user to interact with the 3D object image projected in the air without wearing any special device; (2) the object model (layered cylinder model) is easy to understand following physical laws such as maintaining volume while undergoing large deformation; (3) the interaction method is simplified while resembling actual operations on a real object and is easy to apply to other similar systems. 

Firstly, we propose a layered cylinder model for a 3D object of the pottery on a potter’s wheel. Secondly, we set three kinds of deformation functions to form the object model from an initial state to a bowl shape: shaping the external surface, forming the inner shape (deepening the opening, and widening the opening), and reducing the total height. Next, as for the interaction method between a user and the model, we prepare a simple but similar method to actual hand-finger operations on pottery on a potter’s wheel, in which the index finger movement takes care of the external surface and the total height, and the thumb movement makes the inner shape. Those are implemented in the three-dimensional aerial image interface (3DAII) [1,2,3] developed in our laboratory to build a simulated experience system. We confirm the operation of the proposed object model (layered cylinder model) and the functions of the prepared interaction method (a simple but similar method to actual hand-finger operations) through a preliminary evaluation of participants. 

The remainder of this paper is organized as follows: Section 2 introduces previous works and related studies on the simulated pottery system based on computer technologies and the object/shape model for simulated pottery in order to clarify the positioning of this study; Section 3 explains the object model and the interaction method introduced in this paper; Section 4 presents the three-dimensional aerial image interface (3DAII) as a testing platform in which the object model and the interaction method are implemented, and then it shows and discusses the result of a preliminary evaluation of participants; Section 5 concludes the paper and mentions the remaining issues and future subjects. 

## 2. Previous Works and Related Studies 

### 2.1. Simulated Pottery System 

Various support systems to design and create object shapes using a virtual model based on computer technologies have been researched and developed for decades. Nakano and Watanabe [4] developed a CAD (computer-aided design) system to support a pottery design using sensory glove (cyber glove, data glove) and EM (electro-magnetic) sensors. Korida et al. [5] manufactured a similar system “CHINA (cyber glove-based two-handed interface for virtual artwork environment)” adding a stereoscopic display with liquid crystal (LC) shutter glasses for trial. Both of them have demonstrated that hand motions can operate various deformations of an object model in a virtual environment. 

More and more case studies on hand gesture modeling in a virtual environment have been reported even recently. Unfold Design Studio in Antwerp, Belgium released a “virtual pottery wheel” [6,7] in which a 3D-scanner (laser beam) detects the hand-finger motion to cut away some amount of virtual clay to elaborate its form. Sato et al. [8] used a Kinect sensor (by Microsoft Corp., Redmond, WA, USA) with Zoom for Xbox 360 Kinect (by Nyko Technologies, Los Angeles, CA, USA) to reduce the play range for scraping gestures. Cho et al. [9] adopted an actual spinning wheel as an additional hardware device to present augmented reality to some extent. Krishnamurthy et al. developed “Handy-Potter” [10] and “Shape-It-Up” [11] to propose an idea of shape-gesture-context interplay (SGCI) for natural 3D shape modeling by hand gestures as a natural user interface (NUI) also using a Kinect sensor. Imai et al. [12] developed a cylindrical input device covered with resistive touch sensor sheets to output instructions by means of 3D molding gesture with hands. Krishnamurthy et al. used a Leap Motion Controller (LMC) [13] or a depth sensor [14] equipped with a laptop PC to extract hand motions. Chiang et al. [15] also adopted an LMC to focus on skill training for the general public, providing simulated exercises and problem-solving tasks about pottery. Azuma [16,17] eventually developed a virtual potter’s wheel system “Roquro” also for the general public finally to manufacture a real object using a 3D printer. Most of them used a flat-panel display to provide visual feedback to a user in order to make the system simple, whereas some of them required large-scale facilities or special devices. Han and Han [18] developed a virtual 3D audio-visual interface “virtual pottery” using natural hand motions, and it worked in a special room with a projection on wall surfaces and a motion capture tracking system like OptiTrack (by NaturalPoint, Inc., Corvallis, OR, USA). “DOJAGI” [19,20] is a game software for entertainment to create potteries with the user’s own hands in a virtual reality environment that requires headsets such as the Oculus Rift with Oculus Touch (by Oculus VR LLC, Menlo Park, CA, USA) or the VIVE headset and controller (by HTC Corp., Taoyuan, Taiwan). 

Compared with these works and studies, a system introduced in this paper uses a simple 3D image display (3DAII) without the necessity for each user to wear or put on some special equipment. Therefore, it has the following features: the aerially-projected image can be simultaneously observed from various viewpoints or by multiple users with the naked eye so that a user can directly manipulate the 3D object image by superimposing a user’s hand-finger on the image without wearing any special equipment. 

### 2.2. Object/Shape Model 

The deformation of a 3D virtual object was examined with a simple model like rotationally symmetric objects in the early stages. Sato and Numazaki [21] examined a deformation model of trapezoidal parts as a cross-section of rotational solids while focusing on the constant volume. Kamei et al. [22] discussed the deformation of rotating bodies considering the energy minimization both of the round slice model (strain energy and friction energy) and the contour line model (internal energy of Snakes model [23]). 

Studies on virtual clay were started with the examination of a 3D surface construction algorithm and the development of a modeling interface. Lorensen and Cline [24] proposed a “marching cube” as a high-resolution 3D surface construction algorithm, in which the gradient of original data was found and used as a basis for shading the models. Kameyama [25] developed a deformation support system of a clay model for free-form design, using a 3D mouse and a tactile sensor of I-SCAN75 (by NITTA corp., Osaka, Japan) both with a 3D tracker of FASTRAK space sensor (by Polhemus Company Inc., Colchester, VT, USA). 

Shape modelings were extended to deformations of general clay objects. Arata et al. [26] focused on the cellular automaton to build a 3D discrete active voxel space from interactions in the neighbors, and it led to the distribution and transport of virtual clay following the state transition rules for 3D blocks. McDonnell et al. [27] discussed a virtual clay to realize a natural interface for direct and force-based deformations and developed a sculpting framework by integrating the subdivision solids approach and the physics-based modeling. Knopf and Igwe [28] proposed the self-organizing feature map (SOFM) as a deformable mesh model, and they represented a clay object as hexahedral meshes with nodes (point mass) and springs. Cani and Angelidis [29] reviewed and compared three techniques to deform, add, and remove materials freely in real-time for shape modeling of virtual clay: extension of volumetric implicit sculpting, physically-based deformation, and geometric modeling framework. 

On the other hand, expressions specialized for rotating objects were deepened. Lee et al. [30] presented a circular sector element method (CSEM) that applies a long element method (LEM) [31,32] to pottery with cylindrical symmetry in order to present a model deformation and give some force feedback, calculating a collision detection and a response of a spherical haptic tool for clay deformation. Kumar et al. [33] discussed a kind of number-theoretic approach to create a mathematical model of a rotating object, that is, a digitally connected and irreducible surface of revolution along with a readiness to generate realistic thick-walled potteries. Satoh et al. [8] executed only a linear interpolation of sample points to make lines for outer and inside shapes because they considered only a scrape-off operation by hand gestures. Cho et al. [9] applied the marching cube algorithms [24] for 3D arrays to realize a free-form surface while its surface continuity is maintained. Imai et al. [12] used a software package “Grasshopper” [34], a visual programming language and environment for working on a CAD application “Rhinoceros 3D”, to show a 3D shape including free-form surface. Chaudhury and Chaudhuri [35] introduced cylindrical elements to develop a technique for deformation modeling with volume preservation, however, even after some complicated calculations, various errors in volume preservation remained. Krishnamurthy et al. [14] deformed a virtual pot based on the hand point-cloud (PCL) computing the kernel-density estimation (KDE) and the rate of attraction on the active region of the pot. Chiang et al. [15] applied the Gaussian falloff model for mesh deformation by moving vertices in the center the most and those at the boundaries the least. 

Compared with these works and studies, here we consider a model for a bowl shape, which would be easy to understand and an expression for deformation that should be simple for others to apply. We take care to satisfy three desirable features for virtual clay on physically-based deformation as pointed out in ref [29], as much as possible:

(a) Large scale deformations: the clay will not come back to its initial state after the deformation is applied, which we make possible.
(b) Volume conservation: volume variation should be prevented, which we keep strictly.
(c) Surface tension (to make some effect of deformation operation to the surroundings): the system should mimic the surface tension with the movement of clay referring to physical phenomena, which we express as the influence of the surroundings.

Those are explained in the next section. 

## 3. Methods: Object Model and Interaction Method 

### 3.1. Three-Dimensional Object Model 

**Figure 1:**
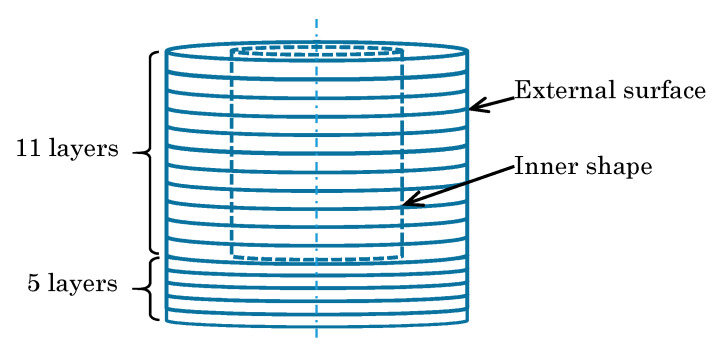
Layered cylinder model for simulated pottery.

The 3D object model introduced here simulating a material (clay) of pottery on a potter’s wheel is a layered cylindrical model stacking sixteen layers of disk-type or ring-type. The initial state of the model consists of the eleven layers of ring-type on the five layers of disk-type, as shown in Figure 1 and Table 1. The number and size of layers were decided by trial and error, considering that the number of layers should be small enough to give a smooth appearance in the testing platform (Section 4). The initial state is set to have a certain amount of hollow in advance, in order to simplify the working process and reduce the working hours to make a bowl shape for a user by interacting with the model. These specific settings should be decided according to the characteristics of the system for implementation and the on-site situations by an application designer or a system integrator. 

### 3.2. Deformation of Model 

We prepared the following three kinds of deformation functions to deform an object model from the initial state to a bowl shape: shaping the external surface, forming the inner shape (deepening the opening, and widening the opening), and reducing the total height. The object model is assumed to rotate on a virtual potter’s wheel, so that the deformation would occur simultaneously all around for diameters and across the whole horizontal plane for heights. 

#### 3.2.1. Shaping the External Surface

**Figure 2:**
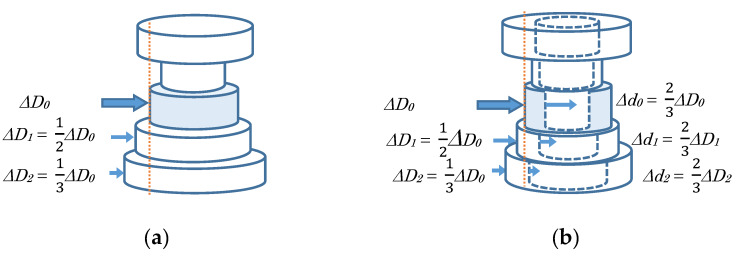
Shaping the external surface: (**a**) in the case of a disk-type layer and (**b**) in the case of a ring-type layer.

- In the case of a disk-type layer (input = Δ*D_0_*) (Figure 2a).
  - At a manipulated layer.
    - External diameter is decreased Δ*D_0_* responding to the manipulated variable, and its height is increased to keep the layer’s volume.
  - At contiguous layers.
    - When its external diameter is larger than the operated layer’s external diameter, it is decreased Δ*D_1_*, Δ*D_2_*, etc. according to a certain rule (e.g., each term of Harmonic series, see Appendix A), and its height is increased to keep the layer’s volume.
- In the case of a ring-type layer (input = Δ*D_0_*) (Figure 2b).
  - At a manipulated layer.
    - External diameter is decreased Δ*D_0_* responding to the manipulated variable, while its inner diameter is also decreased Δ*d_0_* to reduce the layer’s thickness according to the reduction Δ*D_0_* in external diameter (e.g., with an amount of two thirds), and its height is adjusted to keep the layer’s volume.
  - At contiguous layers.
    - When its external diameter is larger than the operated layer’s external diameter, it is decreased Δ*D_1_*, Δ*D_2_*, etc. according to a certain rule (e.g., each term of Harmonic series, see Appendix A), while its inner diameter is also decreased Δ*d_1_*, Δ*d_2_*, etc. to reduce the layer’s thickness according to the reduction Δ*D_1_*, Δ*D_2_*, etc. in external diameter (e.g., with an amount of two thirds), and its height is adjusted to keep the layer’s volume.

#### 3.2.2. Forming the Inner Shape

**Figure 3:**
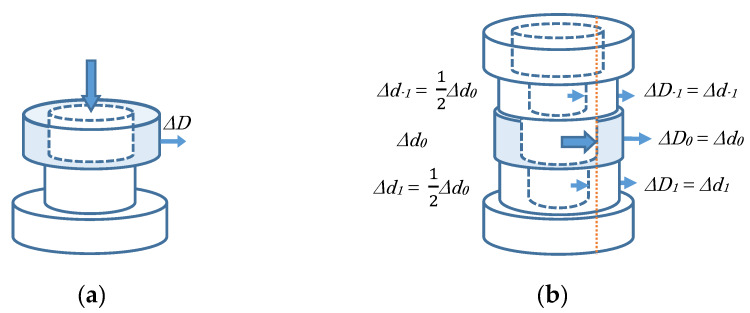
Forming the inner shape: (**a**) deepening the opening and (**b**) widening the opening.

c. Deepening the opening: at a disk-type layer (Figure 3a).
  - At a manipulated layer.
    - An opening is provided with the inner diameter *d* of a certain ratio (e.g., two thirds) to the original external diameter *D*, while keeping its height, and its external diameter is increased Δ*D* to keep the layer’s volume.
d. Widening the opening: at a ring-type layer (input = Δ*d_0_*) (Figure 3b).
  - At a manipulated layer.
    - Inner diameter is increased Δ*d_0_* responding to the manipulated variable, while its external diameter is also increased Δ*D_0_* to keep the layer’s thickness according to the increase Δ*d_0_* in inner diameter (e.g., at the same amount), and its height is decreased to keep the layer’s volume.
  - At contiguous layers.
    - When its inner diameter is smaller than the operated layer’s inner diameter, it is increased Δ*d_1_*, Δ*d_-1_*, etc. according to a certain rule (e.g., each term of Harmonic series, see Appendix A), while its external diameter is also increased Δ*D_1_*, Δ*D_−1_*, etc. to keep the layer’s thickness according to the increase Δ*d_1_*, Δ*d_−1_*, etc. in inner diameter (e.g., at the same amount), and its height is decreased to keep the layer’s volume.

#### 3.2.3. Reducing the Total Height

**Figure 4:**
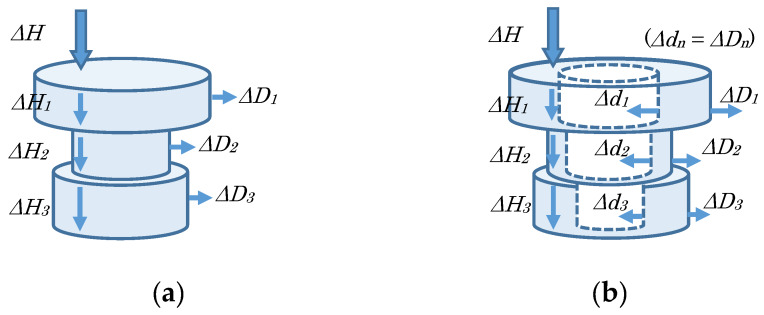
Reducing the total height: (**a**) in the case of a disk-type layer and (**b**) in the case of a ring-type layer.

e. In the case of a disk-type layer (input = Δ*H*) (Figure 4a).
  - All layers.
    - Each height is decreased Δ*H_1_*, Δ*H_2_*, etc. as distributing the manipulated variable Δ*H* according to the original height of each layer *H_1_*, *H_2_*, etc., and its external diameter is increased Δ*D_1_*, Δ*D_2_*, etc. to keep the layer’s volume.
f. In the case of a ring-type layer (input = Δ*H*) (Figure 4b).
  - All layers.
    - Each height is decreased Δ*H_1_*, Δ*H_2_*, etc. as distributing the manipulated variable Δ*H* according to the original height of each layer *H_1_*, *H_2_*, etc., its external diameter is increased Δ*D_1_*, Δ*D_2_*, etc., and its inner diameter is decreased Δ*d_1_*, Δ*d_2_*, etc. (e.g., at the same amount) keeping the layer’s volume.

### 3.3. Interaction Method between User and Model 

In order to design the interaction method between a user and the model, we would keep in mind the following:

- It is supposed that some motion capture sensor can detect and measure the user’s hand-finger movements;
- The user’s hand-finger operations should resemble the actual operations for pottery on a potter’s wheel as much as possible.

Consequently, we have decided to use the index finger and thumb of one hand to perform the three kinds of deformation functions.

**Figure 5:**
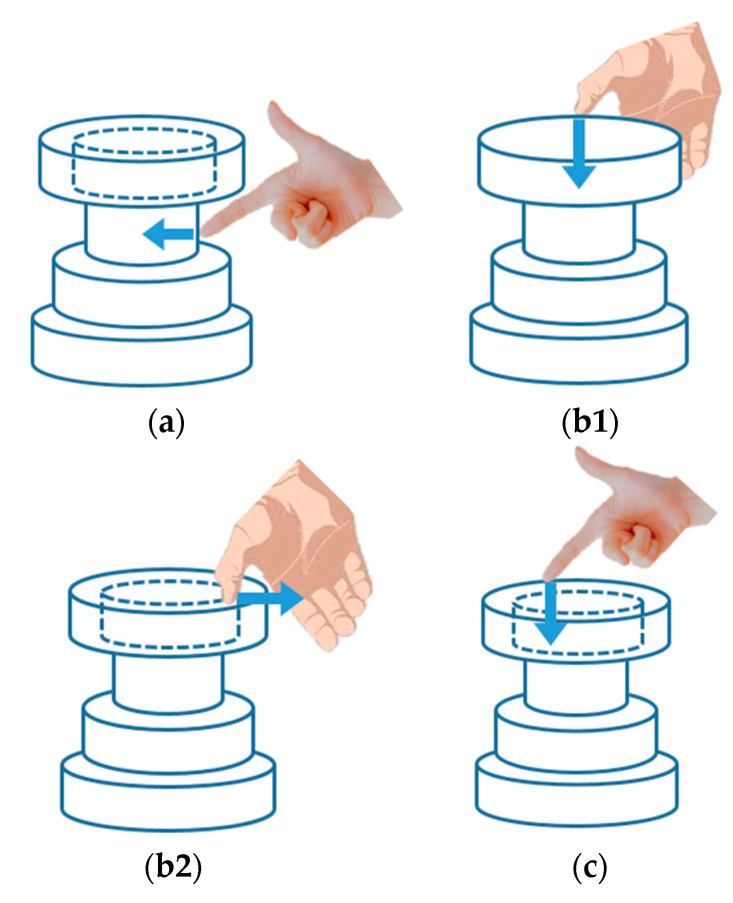
User-model interaction: (**a**) shaping the external surface = index finger; (**b1**) forming the inner shape: deepening the opening = thumb; (**b2**) forming the inner shape: widening the opening = thumb; (**c**) reducing the total height = index finger.

1. Shaping the external surface—index finger.
  - A user puts the index-fingertip to the object model from the outside (Figure 5a).
2. Forming the inner shape—thumb.
  - Deepening the opening.
    - A user puts the thumb-fingertip to the object model from the above (Figure 5b1).
  - Widening the opening.
    - A user puts the thumb-fingertip to the opening of the object model from the inside out (Figure 5b2).
3. Reducing the total height—index finger.
  - A user puts the index-fingertip to the object model from the above (Figure 5c).

Here, the size of the finger is ignored and it is assumed and simplified that the finger is in point contact with the object model because of the small size of the aerially-projected object model in the testing platform (Section 4.1.5). Therefore, only one layer is directly manipulated with the finger at one time based on the end-tip position of a finger measured by a motion capture sensor, regardless of the size of the fingertip. However, since the fingertip has a thickness, it is not a point contact between the fingertip and the object when deforming an object in reality. In addition, when it becomes possible to aerially project an object model at the actual full size of pottery and the model consists of a large number of thin layers for accurate and precise display, it is not always the case that only one layer is directly manipulated corresponding to the thickness of fingertip. In other words, more than one layer should be directly manipulated according to the setting of the object model and the size of the aerially-projected object image. Further, the influence of the single manipulated layer to contiguous layers (Section 3.2) is applied only to the immediate neighboring layer in the testing platform (Section 4), i.e., the immediate neighboring layer is deformed by half of the manipulated layer if the above-mentioned conditions are fulfilled. This influence should also be considered by each designer or integrator depending on the system to implement and the working conditions. 

Please note that the shape deformation is performed based on the concept of speed control. We consider not haptic feedback but visual feedback for users. A user can take his/her fingertips anywhere on the 3D image, regardless of the presence or absence of the 3D object model. If the measurement position of the fingertip is used as the surface position as it should be at that moment for the shape deformation of the object model, the object model can be deformed rapidly and discontinuously regardless of physical laws. Therefore, when the fingertip is placed inside the object surface, the change rate of the surface position is given at a magnitude according to the gap between the surface and the fingertip, in order to prevent an unnatural shape deformation and realize a profile change following physical laws. 

The command value in speed control would become the amount of shape deformation in each control cycle (shape deformation cycle and image update cycle). And this amount of deformation would be Δ*D* in shaping the external surface, Δ*d* in widening the opening, and Δ*H* in reducing the total height. The amount of deformation is calculated by multiplying the gap between the model surface and the fingertip with a gain. A higher gain makes the position of the surface quickly approach the position of the fingertip, and a lower gain makes the position of the surface slowly approach the position of the fingertip. The gain can be adjusted according to the computational power and image update speed of a controller, and it is adjusted by trial and error in the testing platform (Section 4). 

**Figure 6:**
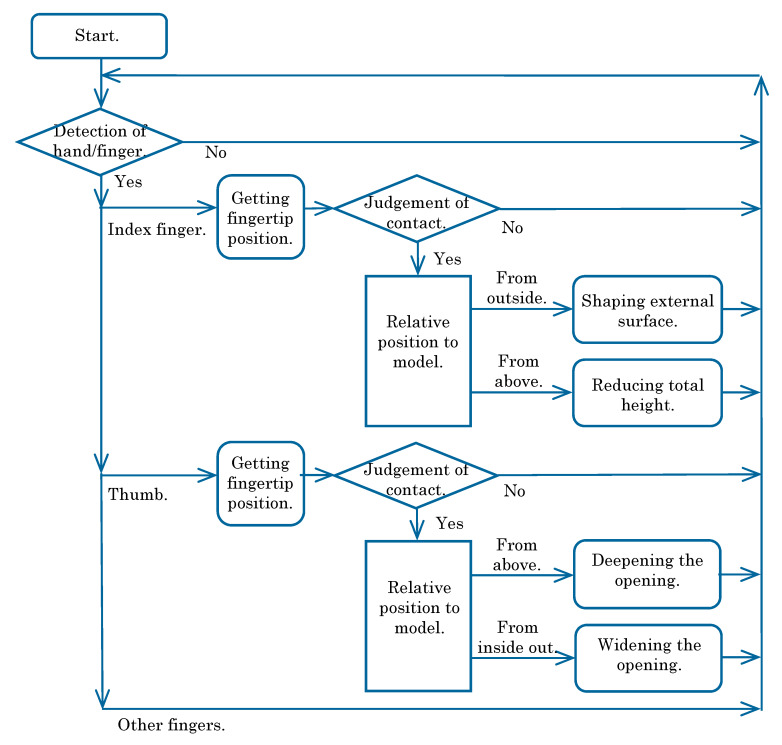
Operation procedure of simulated experience system.

By combining these interactions, a user can deform the object model from the initial state to various bowl shapes. After all, the operation procedure of the simulated experience system of pottery on a potter’s wheel would be expressed as shown in Figure 6. 

## 4. Results and Discussion: Implementation and Preliminary Evaluation 

**Figure 7:**
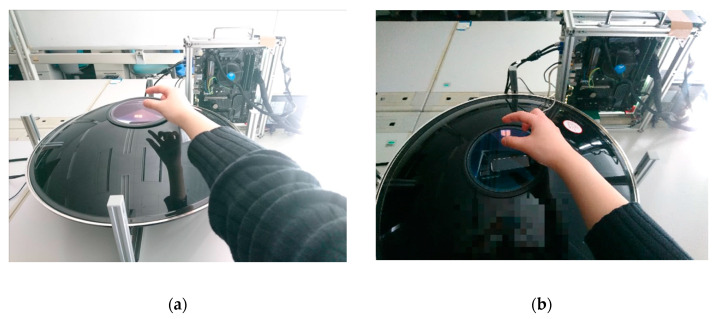
Testing Platform. (**a**) working scene -1, (**b**) working scene -2.

We would like to confirm the operations of the proposed object model (layered cylinder model) (Section 3.1 and Section 3.2) and the functions of prepared interaction method (a simple but similar method to actual hand-finger operations) (Section 3.3) for the simulated experience of pottery on a potter’s wheel. Hence, the three-dimensional aerial image interface (3DAII) [1,2,3] developed in our laboratory was adopted as a testing platform (Figure 7). 

### 4.1. Three-Dimensional Aerial Image Interface (3DAII) 

**Figure 8:**
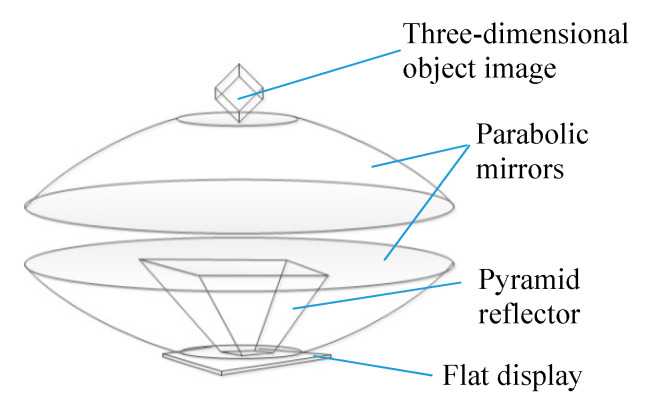
Pyramid reflector and parabolic mirrors.

The 3DAII is an interactive interface in which a user can directly operate an aerially-projected 3D object image (see reference [3]). A pyramid reflector is used to reconstruct the 3D object image, and a pair of parabolic mirrors are used to aerially project the image (Figure 8). A motion capture sensor detects the user’s hand-finger that manipulates the projected object image, and the system immediately exhibits some visual reactions such as deformation, displacement, and discoloration of the object image. Therefore, this interface has several features: a projected image of a 3D object can be simultaneously observed from various viewpoints or by multiple users with the naked eye, and the system comprises simple mechanisms being inexpensive to realize and easy to install and relocate. 

#### 4.1.1. Pyramid Reflector 

Four kinds of side view (front, rear, right side, and left side) are prepared. Those four images/videos are displayed in the appropriate position and orientation on the flat-panel display. A pyramid composed of transparent material like acrylic resin is set on the flat-panel display, and then the side inclined plane of it becomes a tilted beam splitter (half mirror). When an observer looks into the pyramid from its side, he/she feels the presence of a 3D object inside the pyramid with depth and stereo feeling, although he/she watches only two-dimensional image/video. A design example of the pyramid reflector is shown in [3], and specifications of the pyramid reflector used in the testing platform are indicated in Table 2. 

#### 4.1.2. Parabolic Mirrors 

Two concave mirrors are set to face so as to make those rims coincide with each other, and then it is possible to show that an object close to the focal point of one concave mirror seems to be close to the focal point of the other concave mirror. When we put an object close to the focal point of the upper concave mirror (at the bottom portion of the parabolic mirrors), the light from the object reflected by the upper and lower mirrors is focused to make an image close to the focal point of the lower concave mirror (at the ceiling portion of the parabolic mirrors). Therefore, when we watch the device from obliquely above, the object at the bottom portion is visible as if it exists in the air close to the opening part of the device. The specifications of the parabolic mirrors used in the testing platform are indicated in Table 3. The projection area of a 3D object image would become small considering the structure of the pyramid reflector and the diameter of two circular opening parts of parabolic mirrors; practically, such as a 30-mm cube is an example. 

#### 4.1.3. Motion Capture Sensor 

The motion capture sensor detects the operational state of a user’s hand-finger which would be used as an input to take a system reaction. We adopted Leap Motion Controller (LMC) by Ultraleap Ltd., Bristol, UK (Table 4), which serves our purpose with a large working area (2.5–60 cm in distance, 150 deg in viewing angle), high frame rate (200 fps at maximum), and high precision (1/100 mm at maximum). 

**Figure 9:**
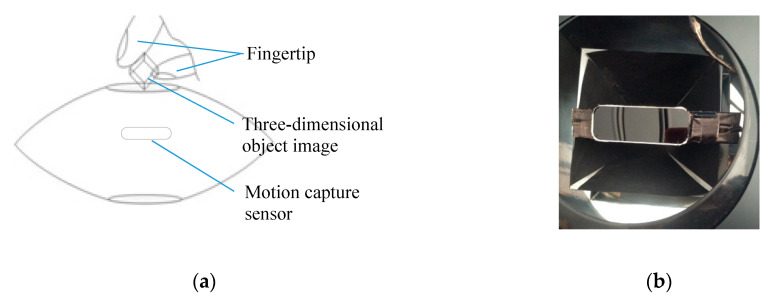
Setting of motion capture sensor: (**a**) longitudinal section; (**b**) top view.

LMC is set inside the pyramid reflector located in the parabolic mirrors as it looks up (Figure 9). While doing the pottery on a potter’s wheel, a user usually stretches forth his/her hand-finger and make them approach from above the clay object. When LMC is set in the front or in the rear of the aerially-projected object image to detect the user’s hand-finger, it hardly detects them because the palm or the dorsum of the hand cannot be seen clearly from the LMC. Even if LMC is set at the right-side or left-side of the object image, it is difficult to recognize the user’s hand-finger correctly because the five fingers occlude each other. If LMC is installed above the object image to look down the user’s hand-finger, it still cannot measure them correctly because it is influenced by the projection light from below. Consequently, LMC is placed below inside the parabolic mirrors to measure the user’s hand-finger as it looks up. Although a usual calibration procedure (correspondence between the coordinate system of LMC and the coordinate system in computer graphics) is necessary according to the location of LMC, no special consideration is required. However, depending on the installation location of the system, ceiling lights would have a bad influence on the measurement performance of LMC. In such a case, some black canopy would be provided to make the measurement performance better, which also improves the visibility of the aerially-projected object image for users. 

#### 4.1.4. Software 

Software to work the testing platform was developed on Windows PC (CPU: Intel (R) Core (TM) i5-7200U @ 2.50 GHz (Intel Corp., Santa Clara, CA, USA), embedded GPU: Intel High-Definition (HD) Graphics 620, RAM: 8GB) by C# programming language using MS Visual Studio as IDE (integrated development environment). While creating an execution file, Unity (ver.5.6.1f1) for modeling and drawing 3D objects and Leap Motion SDK (v2.3.1) (Ultraleap Ltd., Bristol, UK) for measuring hand-finger movements were used as a program library. 

#### 4.1.5. Implementation Results 

The 3DAII used as a testing platform aerially projects a 3D image as follows:

- The initial state of the object model is 25.0 mm in diameter and 5.8 mm in height as external appearance, and 16.7 mm in diameter and 4.6 mm in depth as hole-size;
- The inner shape is presented in lower translucent while the external surface is presented in higher translucent;
- A user’s fingertips detected by LMC are displayed as a dot in green for the index finger and a dot in red for the thumb as the marker of the operating part;
- The layer of the object model that a user is processing is displayed in a darker color as the manipulated layer under processing.

The most distinctive feature of the simulated experience system of pottery on a potter’s wheel which is examined in this paper is that a user can directly manipulate the aerially-projected object image by superimposing his/her own hand-finger on the image. However, the projection area of 3DAII used as a testing platform (Section 4.1.2) and the size of the aerially-projected object image are small as mentioned above so that the operation area for the user’s fingertip is set at about 30 mm right above the projection area of object image to prevent occlusions (invisible portions of object image hidden behind user’s hand-finger). The colored dot is a marker of the operating part that manipulates the layer in a darker color of the object model while processing. 

**Figure 10:**
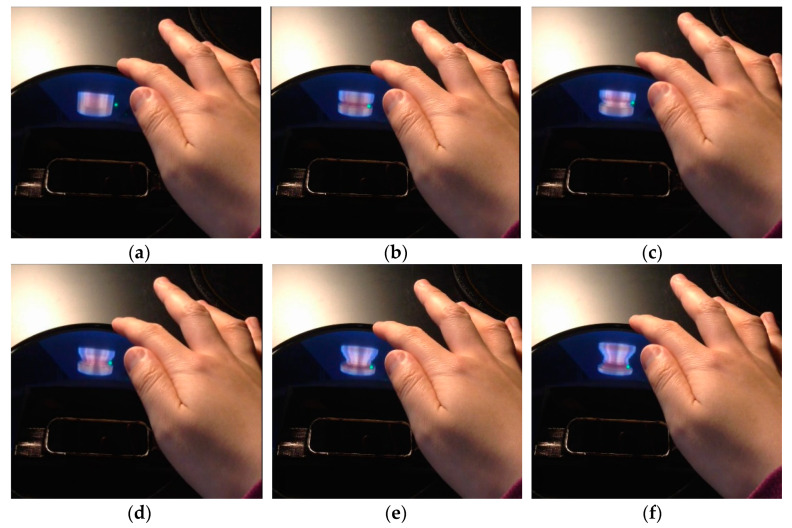
Shaping the external surface: (**a**) → (**b**) → (**c**) → (**d**) → (**e**) → (**f**).

**Figure 11:**
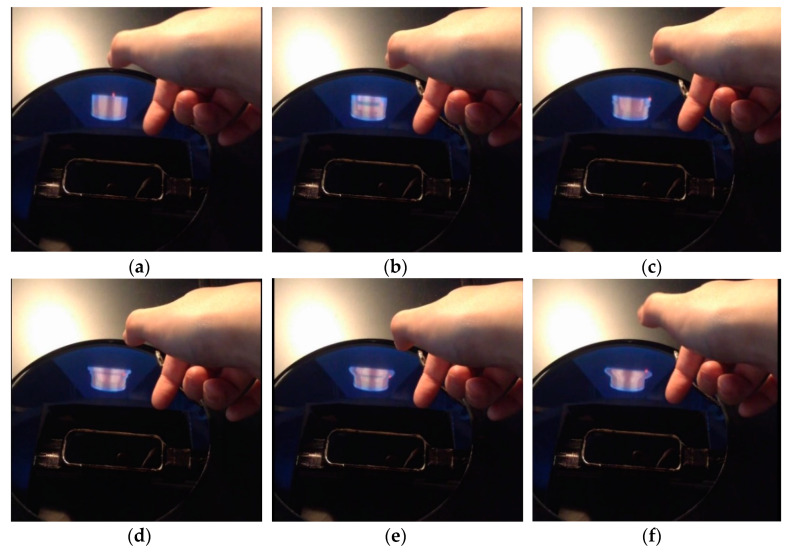
Forming the inner shape: (**a**) → (**b**) → (**c**) → (**d**) → (**e**) → (**f**).

**Figure 12:**
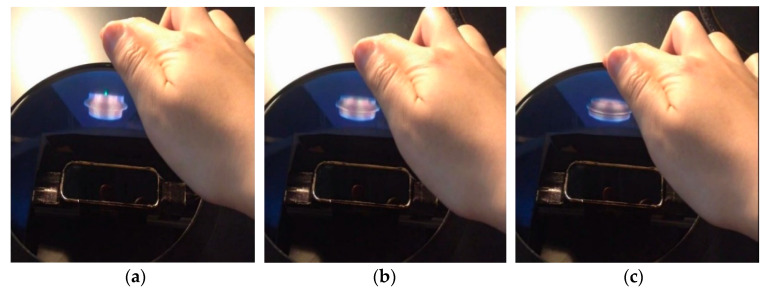
Reducing the total height: (**a**) → (**b**) → (**c**).

In this way, we realized the three kinds of deformation functions to form the object model from an initial state to a bowl shape as shown in Figure 10, Figure 11 and Figure 12.

### 4.2. Preliminary Evaluation 

#### 4.2.1. Purpose 

The purpose of this preliminary evaluation is to confirm that the system allows even a beginner to make the object model (layered cylinder model) deform from the initial state to various bowl shapes by combining finger–model interactions (a simple but similar method to actual hand-finger operations) for three kinds of deformation functions. 

#### 4.2.2. Procedure 

The preliminary evaluation of participants was conducted as follows:

1. A participant received an explanation about the deformation function of object mode (watching Figure 2, Figure 3 and Figure 4) and the interaction method with the object model (watching Figure 5);
2. A participant performed random operations to familiarize himself/herself with the simulated system and its operations, and practiced what he/she had been explained until when he/she was satisfied to some extent;
3. A participant was asked to make three kinds of bowl shapes according to the sample indicated in Table 5: cylindrical, dome-shaped, and flat type, so that he/she would be able to understand features of each bowl shape;
4. A participant answered the survey on maneuverability, visibility, and satisfaction (including presence and accomplishment).

The participants are twelve master course students (right-handed) who are unrelated to this research project but have enough capacity to understand instructions to try their best to make a bowl shape and express opinions about this simulated experience system. Most of the participants were inexperienced in pottery making with a wheel, however, the programmer (second author) of this simulated experience system is experienced and she has developed the program based on a real experience. This preliminary evaluation aims at confirmation of the operation of the proposed object model (layered cylinder model) and the functions of the prepared interaction method (a simple but similar method to actual hand-finger operations) so that a comparative study between experienced and inexperienced is not conducted, and a quantitative evaluation about the workmanship of bowl shape created by participants is not made. 

#### 4.2.3. Result 

(A) Making bowl shapes 

All participants could make something like three kinds of bowl shapes in less than 30 min since their first touch. Examples of participant’s work are shown in Table 6. 

• Cylindrical bowl: 

The depth of opening would be insufficient only by shaping the external surface. When the opening is deepened, the external surface is also deformed. Therefore, some trial and error is required. All the participants could realize the straight external surface and the bottomed-out inner shape as the feature of a cylindrical bowl to some extent. 

• Dome-shaped bowl: 

Also, in this case, some trial and error is required because it would be insufficient to form the opening when having attention caught by the typical feature of rounded external surface too much. Although, all the participants could realize it to some extent. 

• Flat-type bowl: 

Some skillful operation for the inner shape is required to widen the opening with the feature of an inclined external surface. This was difficult for most participants as that they had to get used to the testing platform with the translucent display and the fingering technique for processing. 

(B) Questionnaire survey 

**Figure 13:**
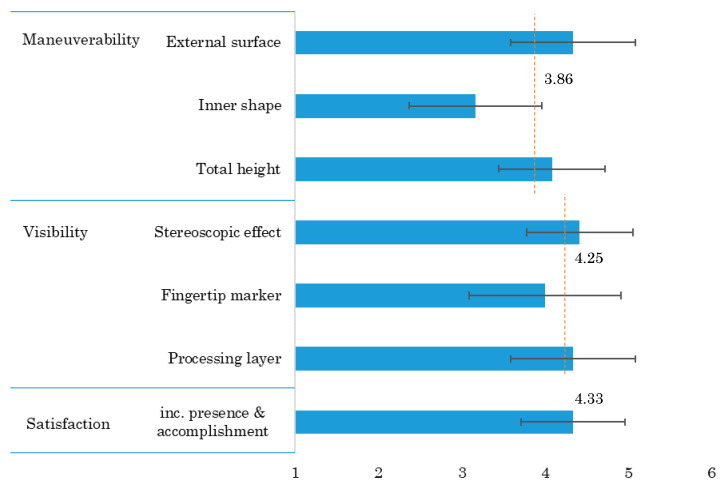
Result of questionnaire survey (*n* = 12).

The participants evaluated maneuverability, visibility, and satisfaction on a five-point Likert scale (1: very poor, 2: poor, 3: fair, 4: good, 5: excellent) and left their opinions and suggestions in the free entry field. The result of the questionnaire survey is shown in Figure 13. 

• Maneuverability: 

The score indicates the operation for intuitive modeling to make a bowl shape. We asked for a “good” or “poor” review for each of three deformation functions: shaping the external surface, forming the inner shape (both deepening and widening the opening), and reducing the total height. The average (Av) with standard deviation (SD) of evaluation score for overall maneuverability is 3.9 ± 0.9, showing as almost good. The evaluation score is high on the function of shaping the external surface (4.3 ± 0.7) and that of reducing the total height (4.1 ± 0.6), meanwhile the score is low on the function of forming the inner shape (3.2 ± 0.8). In the free entry field, there were the following opinions about maneuverability.

○ (External surface and inner shape) “The function of shaping external surface is good, but it is a little difficult to control forming inner shape.”
○ (Height) “The reducing height function is easy to be misrecognized as the external surface function.”
○ (Processing speed) “The processing speed is relatively slow, but thanks to that, it is easy to make fine-tuning.”

• Visibility: 

The score assesses the appearance in an aerially-projected image. We asked for a “good” or “poor” review on the stereoscopic effect and the indication of the operating part (fingertip marker) and processing part (manipulated layer). The Av with SD of evaluation score for overall visibility is 4.3 ± 0.8, showing as good enough. The evaluation score is high all on the stereoscopic effects (4.4 ± 0.6), the indication of the fingertip marker (4.0 ± 0.9), and the indication of the manipulated layer (4.3 ± 0.8). The feedback about visibility was as follows.

○ (Positive) “The visibility is good”, and “the displaying is easy to understand”.
○ (Negative) “The view direction is limited, for some tall people they need to lower their head to be sure to see the object”, and “we should feel more in three-dimensional effect”.

• Satisfaction: 

The score represents the comprehensive evaluation including the presence of the pottery on the potter’s wheel and the accomplishment as a simulated experience. The Av with SD of evaluation score for satisfaction is 4.3 ± 0.6, showing as pretty good. In the free entry field, we received some dissatisfactions to consider at various levels, from individual functions to design policies, along with favorable reputations.

○ (Sensing) “The sensitivity of fingertip changes according to the distance to hand-finger so that it is hard to control”, and “the sensitivity can be improved in the future work”.
○ (Cancel/undo function) “It should provide some skip back function to cancel the mistake operation.”
○ (Guidance) “If there is some guidance of how it can be operated more, that will be better.”
○ (Practice) “Although it was somewhat difficult for new users, but once you have catch the method to control it, you will enjoy it very much.”
○ (Fatigue) “A long time use makes my hand/arm tired”, and “to prevent my arm hurt, some support should be settled”.
○ (Positive, specific) “The total comprehension of project is good”, “I think it’s a very interesting innovation”, “the system is special and interesting”, and “this device works well and can help the person who wants to learn pottery”.
○ (Positive, general) “We can use this device to operate for intuitive modeling”, and “in the future with upgrading this device can be marketization”.

#### 4.2.4. Discussion 

• Object model and interaction method: 

The effect of combining the proposed object model (layered cylinder model) with the prepared interaction method (a simple but similar to actual hand-finger operations) appears in the “maneuverability”. Even though the SD of evaluation score for maneuverability is relatively large and it shows that the individual difference both on learning ability and manual dexterity is presented especially for beginners in this system, it can be said that the layered cylinder model proposed here is enough to deform and make a bowl shape according to the participants’ performance. However, it is difficult for some participants to form the inner shape and reduce the total height because the relatively-thick thumb is used to make the inner shape and the single index-fingertip operates two different functions for external surface and total height. As mentioned in Section 4.1.5, the size of the aerially-projected object image is small so that it has been a little difficult for some participants to see the narrow opening of the object model while processing. It will be solved to some extent by increasing the size of the display device (3DAII). Some participant’s feedback shows the intended effect such that the shape deformation is executed based on the concept of not position but speed control, as mentioned in Section 3.3. This point is an important feature of the shape deformation prepared for the proposed layered cylinder model and one of the technical contributions of this study, which is not explicitly shown in the conventional user interactions with object images. 

• Display capability of 3DAII: 

The display capability of 3DAII used as a testing platform is shown in the “visibility”. Even though the SD of evaluation score for visibility is large, showing that the recognition of displaying by 3DAII varies among individuals, stereoscopic displaying by 3DAII has been accepted by participants to some extent. In addition, utilizing the characteristics of the virtual system, some participants’ positive comments show that the desired effect has been obtained by displaying both the fingertip markers as the operating part and the manipulated layer as the processing part. Some negative comments also indicate the limitation of 3DAII’s performance. 3DAII uses a type of illusion technique similar to Pepper’s ghost in which although a user only watches two-dimensional images/videos he/she feels the presence of a three-dimensional object with a depth and stereo feeling. 

• Simulated experience system of pottery on a potter’s wheel: 

The practicality and usefulness of the simulated experience system of pottery on a potter’s wheel constructed here is shown in the “satisfaction” (comprehensive evaluation including presence and accomplishment). According to the evaluation score for satisfaction and some participants’ feedback, this simulated experience system is generally well-received by participants and the system might be easier accepted by younger generations. We need to ask a wide variety of people to make a trial of this system in order to get wider range of opinions as feedback. It would be possible to have many people experience the system in a trial as a technology demonstration, for example, at science museums. Of course, it is difficult to commercialize the current system as it is because several issues should be considered. Sensor technology always faces challenges in reliability and accuracy even under bad conditions such as the presence of disturbance light, and so we would like to try other sensors rather than LMC to capture a user’s hand-finger movements. It depends on the system design concept whether a cancel/undo function to cancel the last operation should be prepared or not. It depends on whether the target system is for entertainment or for experience, and here we choose the latter. In our system, a user will not necessarily become rapidly skillful at doing the work. We think the system should provide a real experience, which includes one where a user does not work well, one where the process does not go the way he/she wants, and sometimes failure. If a recovery after a failure goes wrong, a user can start again from the beginning in an experience system. In addition, we would like to intend a system which is easy even for beginners to use fully after getting used to it by trial and error without some operations manual or guidance. The advantage of a simulated experience system is that a user can start over and over again if he/she does not spare time and effort. It requires trial and error as practices until mastering something, therefore, it is also another subject to make the system so that a user can enjoy the practicing process. Furthermore, fatigue and tiresomeness are common problems for experience/edutainment systems. We also have to consider the user’s operation posture. 

• Utilization and generalization of findings: 

This paper examined an object model for the bowl shape (layered cylinder model) which would be easy to understand and an expression for deformation of the model that should be simple for others to apply. Various models and deformations have been proposed so far (Section 2.2), therefore, system designers and application developers would be able to choose their appropriate model and method considering our proposal according to their purpose and priority factors. Regarding the interaction method between a user and an aerially-projected 3D object image, we prepared a simple but similar method to actual hand-finger operations, but the so-called standard technique would not be suddenly established. A steady approach aiming at generalization can be considered by extracting essences from each result while repeating case studies. This study shows that the simulated experience of pottery on a potter’s wheel is an appropriate application taking advantage of the features of 3DAII. We would like to consider other applications such as an interface of a center console (control panel) for automobiles. 

## 5. Summary and Conclusions 

This paper introduced an object model and an interaction method for a simulated experience of pottery on a potter’s wheel. Firstly, we proposed a layered cylinder model for a 3D object of the pottery on a potter’s wheel. Secondly, we set three kinds of deformation functions to form the object model from an initial state to a bowl shape: shaping the external surface, forming the inner shape (deepening the opening, and widening the opening), and reducing the total height. Next, as for the interaction method between a user and the model, we prepared a simple but similar method to actual hand-finger operations on pottery on a potter’s wheel, in which the index finger movement takes care of the external surface and the total height, and the thumb movement makes the inner shape. Those were implemented in the three-dimensional aerial image interface (3DAII) developed in our laboratory to build a simulated experience system. We confirmed the operation of the proposed object model (layered cylinder model) and the functions of the prepared interaction method (a simple but similar method to actual hand-finger operations) through a preliminary evaluation of participants. The participants were asked to make three kinds of bowl shapes (cylindrical, dome-shaped, and flat-type) and then they answered the survey (maneuverability, visibility, and satisfaction). All participants could make something like three kinds of bowl shapes in less than 30 min from their first touch. The Av and SD of evaluation score on the five-point Likert scale (1 to 5) were 3.9 ± 0.9 for maneuverability, 4.3 ± 0.8 for visibility, and 4.3 ± 0.6 for satisfaction. From the result of the questionnaire survey (evaluation score and opinions of participants) in the preliminary evaluation of the simulated experience system of pottery on a potter’s wheel constructed here as a case study, it was suggested that the design concept, in which a user directly superimposes his/her hand-finger on an aerial-projected three-dimensional image without special conditions such as mounting equipment and environmental preparation, could make a positive contribution to the human-system interaction in a certain practical application, along with the proposed model (layered cylinder model) and the prepared interaction method (a simple but similar method to actual hand-finger operations). 

There are several remaining issues and future subjects for the simulated experience system of pottery on a potter’s wheel based on the discussion in Section 4.2.

- Larger-scale displaying:
  We have confirmed the proposed design concept of projecting a small-sized object model using 3DAII as a testing platform, but we should prepare a larger-sized 3DAII to project a model at actual size to achieve exact overlapping of a user’s hand-finger with an object model.
- Functionality improvement:
  Although we have realized three kinds of deformations as the minimum-required function to make a bowl shape, we should prepare additional functions to bring the simulated experience closer to a real work, such as removing some part of the material without keeping its volume and painting after making a bowl shape.
- Various users:
  We should ask a variety of participants, not only young adults to try to use the simulated system to obtain various feedback.
- Detailed and rigorous evaluation experiments and results analysis:
  We would like to consider a detailed and rigorous evaluation and analysis, if the value and effectiveness of the simulated experience system constructed here are recognized, even though it is difficult to compare with other systems with different design concepts and system configurations.

## Figures and Tables

**Table 1:** Initial state of layered cylinder model.

	Model Size (in “Unit” by Unity ^1^)	
	Diameter	Height
Disk-type layer (single)	1.5	0.014
Ring-type layer (single)	1.5 (external), 1.0 (inner)	0.025
Total (16 layers)	1.5	0.345

^1^ A cross-platform game engine developed by Unity Technologies (see Section 4.1.4).

**Table 2:** Specifications of a pyramid reflector.

Item	Specification
Material	Acryl (mirror-finished coating)
Shape	Inverted pyramid
Width	14 (upper side), 0 (bottom side) cm
Height	12 cm

**Table 3:** Specifications of parabolic mirrors.

Item	Specification
Product	Giant Mirage (Opti-Gone International Inc.)
Material	Acryl (mirror-finished coating)
Diameter	55.9 (external), 15.2 (hole) cm
Height	15.2 cm

**Table 4:** Specifications of motion capture sensor.

Item	Specification
Product	Leap Motion Controller (Ultraleap Ltd.)
Size	W80, D30, H11 mm
Interface	USB 2.0/3.0
Method	Infrared LED (3) + Monochromatic IR sensor (2)
Tracking speed	120 (150) fps (balance mode), 60 (80) fps (precision mode), 214 (295) fps (high speed mode)
Interaction box	W235, D147, H235 (82.5–317.5) mm

**Table 5:** Sample of bowl shapes.

Item	Cylindrical	Dome-Shaped	Flat-Type
Appearance	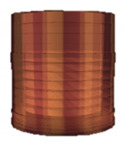	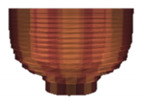	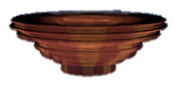
Height	High	Normal	Normal
External surface	Waist-less, Straight	Sweeping, Rounded	Inclined, Slightly-curved
Inner shape	Straight, Bottomed-out	Rounded, Bottomed-out	Inclined, Bottomed-out

**Table 6:** Examples of participant’s work.

Item	Cylindrical	Dome-Shaped	Flat-Type
Participant A’s work	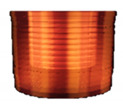	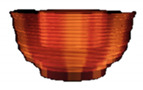	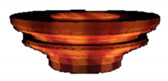
Participant B’s work	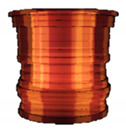	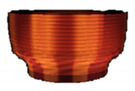	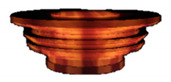

**Figure A1:**
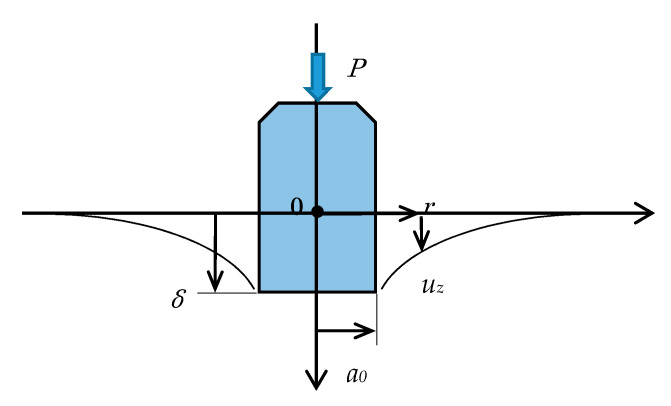
Surface deformation of a perfectly elastic body by an indentation of a circular flat punch.

## Appendix A. Shape of a Solid Surface When an Indenter Is Pushed

We consider the shape of a solid surface when an indenter is pushed. The indentation contact mechanics is positioned as a contact problem treated by continuum mechanics. When a load (force) is applied to a certain solid, some deformation is generated as a mechanical response. For simplicity, we discuss the surface deformation in the case that a circular flat punch is indented on a semi-infinite perfect elastic body in which stress and strain are proportional (Figure A1).

Constants and variables are set as follows:

Indentation load *P*

Young’s modulus *E*

Poisson’s ratio $\nu =\frac{-\varepsilon_{radial}}{\varepsilon_{axial}}$

($\varepsilon_{axial}$: Axial stress, $\varepsilon_{radial}$: Radial stress)

Plane strain Young’s modulus $E^{\prime }=\frac{E}{1-\nu^{2}}$

Non-dimensional radial distance $\rho =\frac{r}{a_{0}}$

(*r*: Distance from indentation axis, *a_0_*: Contact radius)

Then, the indentation depth and the deformation of solid surface according to the indentation load are expressed as the following equations [36,37]:

Indentation depth $\delta =\frac{1}{2E^{\prime }}\frac{P}{a_{0}}$

Displacement of free surface $u_{z}=\frac{1}{\pi E^{\prime }}\frac{P}{a_{0}}\sin^{-1}\frac{1}{\rho }=\frac{2\delta }{\pi }\sin^{-1}\frac{1}{\rho }$

Therefore, the relationship between the indentation depth and the displacement of solid surface according to the distance from the indentation axis is as follows:

$∴\frac{u_{Z}}{\delta }=\frac{2}{\pi }\sin^{-1}\frac{1}{\rho }$

The indenter is corresponding to an input for shaping the external surface or widening the opening in Section 3.2. Therefore, the indentation depth *δ* is equivalent to the displacement input at the manipulated layer: Δ*D_0_* (for shaping the external surface) or Δ*d_0_* (for widening the opening).

Several numerical examples of the relationship between the non-dimensional distance and the relative displacement are indicated in Table A1. It can be seen that the values of the relative displacement are close to those of each term of the Harmonic series. Therefore, in the system explained in this paper, we adopted the terms’ value of Harmonic series on trial to express some influence on the neighbors from the manipulated part.

Specifically, we would like to discuss the relationship between the indentation depth and the solid surface deformation, i.e., the relationship between the position of the fingertip and the deformation of the object model. As introduced in Section 3.1, the solid object is modeled as stacked layers. The influence of the single manipulated layer to the contiguous layers (Section 3.2) is applied only to the immediate neighboring layer in the testing platform as explained in Section 3.3. However, in general, even if the degree of deformation would be set according to the terms’ value of Harmonic series, it is necessary to examine the deformation of not only the manipulated layer but also the contiguous layers, such as which layer should be deformed and how much it would be deformed, depending on the size of the fingertip and the number and height of layers forming the object model.

Furthermore, although the above-mentioned equations describe the case of a perfectly elastic body for a solid object, the clay for pottery is at least, a viscoelastic body. In addition, the above-mentioned equations describe the case of indentation in a flat surface, but the clay surface would be almost bumpy/uneven while working on pottery on a potter’s wheel. Those factors cause deviations from the approximation of the equations.

**Table A1:** Relationship between distance and displacement.

Non-Dimensional Distance *ρ*	Relative Displacement $\frac{u_{Z}}{\delta }$	Terms of Harmonic Series
1	1	1
1.5	0.46	1/2 = 0.5
2	0.33	1/3 = 0.333
2.5	0.26	1/4 = 0.25
3	0.21	1/5 = 0.2
3.5	0.18	1/6 = 0.166
